# Evaluation of the relationship between pan-immune inflammation value and prognostic factors in germ-cell testicular cancer: analysis of stage, lymph node involvement, and metastasis

**DOI:** 10.55730/1300-0144.6135

**Published:** 2025-11-17

**Authors:** Erdinç DİNÇER, Orkunt ÖZKAPTAN, Cengiz ÇANAKÇI, Osman Murat İPEK, Alper COŞKUN, Ahmet Halil SEVİNÇ

**Affiliations:** Department of Urology, University of Health Sciences, Kartal Dr. Lütfi Kırdar City Hospital, İstanbul, Turkiye

**Keywords:** Pan-immune inflammation value, germ cell tumors, testicular tumors

## Abstract

**Background/aim:**

To evaluate the association between the preoperative pan-immune inflammation value (PIV) and prognostic factors, including stage, lymph node involvement, and metastasis, in patients who underwent radical orchiectomy for germ cell testicular tumors.

**Materials and methods:**

Data from 178 patients who underwent radical orchiectomy for testicular tumors between January 2014 and January 2024 were retrospectively reviewed. Preoperative serum tumor markers, hematologic parameters, and histopathological findings were recorded. Tumor staging was determined based on radiological imaging and serum tumor markers. The PIV was calculated using the formula: (monocyte count × neutrophil count × platelet count) / lymphocyte count. Optimal cut-off values for PIV were determined using ROC curve analysis. The association between the PIV score and clinicopathological variables was analyzed.

**Results:**

The mean age of the patients was 32.34 ± 9.62 years. The mean PIV score in patients with metastasis and retroperitoneal lymph node invasion (RPLNI) was significantly higher than in those without metastasis or RPLNI (p = 0.01 and p = 0.04, respectively). The PIV score increased progressively with higher tumor T, N, and M stages. Additionally, a statistically significant increase in PIV scores was observed among patients classified into higher International Germ Cell Cancer Collaborative Group risk groups (p = 0.01).

**Conclusion:**

Higher PIV scores are significantly associated with tumor stage, lymph node involvement, and metastasis in patients with germ cell testicular tumors. The PIV score appears to be a useful and cost-effective preoperative marker for predicting advanced disease in testicular tumors at the time of diagnosis.

## 1. Introduction

Testicular malignancies account for approximately 1% of all cancers in men [1]. Nonetheless, it represents the most common solid malignancy among males aged 20 to 35 years [2]. In developed countries, the prevalence of testicular cancer is approximately 5.7 per 100,000 and studies have shown that this rate has been increasing over time [3,4]. Germ cell tumors account for 90%–95% of all testicular neoplasms. Although the overall mortality rate of testicular tumors is low, they can cause significant socioeconomic and psychological challenges for young men. The 5 year survival rate has been reported to be approximately 97% [5,6].

Human chorionic gonadotropin (hCG), alpha-fetoprotein (AFP), and lactate dehydrogenase (LDH) are routinely used for both diagnosis and follow-up. These biomarkers are essential for diagnosis; however, they have limited sensitivity and specificity [7]. Prognosis primarily depends on factors such as stage at diagnosis and the presence of metastasis, despite the overall high survival rate in testicular cancer [2]. For these reasons, novel diagnostic and prognostic biomarkers are being explored.

Numerous studies have demonstrated that inflammation plays a significant role in cancer development and progression [8]. Lymphocytes promote apoptosis in tumor cells by mediating cytotoxic immune responses [8]. In contrast, neutrophils and monocytes constitute key components of the innate immune defense against carcinogenic stimuli [8]. A delicate balance exists among these immune cell populations; when disrupted in favor of tumor progression, neutrophils, monocytes, and platelets may contribute to tumor growth and dissemination [8]. Previous studies have shown that cancer-associated macrophages derived from monocytes, together with neutrophils and platelets, facilitate primary tumor expansion, invasion, and distant metastasis [8]. Based on this evidence, inflammatory indices such as the neutrophil-to-lymphocyte ratio (NLR), platelet-to-lymphocyte ratio (PLR), hemoglobin–albumin–lymphocyte–platelet (HALP) score, and systemic immune-inflammation index (SII) have been developed to predict prognosis in various malignancies, and their prognostic significance has been validated.

The pan-immune inflammation value (PIV) is a recently proposed biomarker, and studies have demonstrated its superiority over other inflammatory indices in predicting prognosis in metastatic colorectal cancer [9,10]. Studies have also indicated that PIV is effective in predicting prognosis in other urological malignancies, including bladder and prostate cancers [11,12]. However, the association between PIV and testicular tumors has not yet been investigated in the existing literature. Therefore, we aimed to evaluate the association between PIV and prognostic factors, including stage, lymph node involvement, and distant metastasis, in patients with germ cell testicular tumors.

## 2. Materials and methods

The study was approved by the Institutional Ethics Review Board (approval no: 2025/010.99/12/26; approval date: 24 January 2025). Medical records of 256 patients aged 18 to 65 years who underwent radical orchiectomy for testicular tumors between January 2014 and January 2024 were evaluated. Patients who were not pathologically diagnosed with germ cell tumors (n = 48), had a history of other malignancies (n = 9), had missing data, or presented with conditions affecting inflammatory markers—such as immunosuppressive disorders, medication use, or active infections—(n = 21) were excluded. Consequently, 178 patients with testicular tumors pathologically confirmed as germ cell tumors were included in the study.

Patient age, histopathological findings, preoperative and postoperative PIV values, tumor markers, and radiological imaging results were recorded. The PIV was calculated using the following formula: (monocyte count × neutrophil count × platelet count) / lymphocyte count, expressed in 10^9^/L. Clinical staging was performed based on contrast-enhanced abdominal and thoracic computed tomography obtained the day before surgery. Patients were classified according to the TNM classification system, tumor stage, and International Germ Cell Cancer Collaborative Group (IGCCCG) risk groups. The S stage was evaluated according to serum tumor markers measured during the second postoperative week. Postoperative follow-up was conducted according to the European Association of Urology guidelines [13]. The relationships between the PIV score and tumor stage, prognosis, lymph node involvement, and metastasis were analyzed. Written informed consent was obtained from all participants prior to study enrollment.

### 2.1. Statistics

Statistical analyses were performed using SPSS 21.0 (IBM Corp., Armonk, NY, USA). Skewness and kurtosis were calculated to assess the normality of the data distribution. Results were presented as medians and interquartile ranges (IQR) for nonnormally distributed data. Nonnormally distributed variables were analyzed using the Mann–Whitney U test and the Kruskal–Wallis test. Median (IQR) values were used to describe continuous variables with nonnormal distribution. Categorical variables were expressed as numbers and percentages. The prognostic performance of PIV was evaluated using receiver operating characteristic (ROC) curve analysis and the corresponding area under the curve (AUC). Statistical significance was set at a p value of <0.05.

## 3. Results

The study included a total of 178 male patients. The mean age of the patients was 32.34 ± 9.62 years, with a median of 31 (range: 17–61). Histopathological evaluation revealed 90 seminomas (50.6%), five embryonal carcinomas (2.8%), 74 mixed germ cell tumors (41.6%), one yolk sac tumor (0.6%), seven teratomas (3.8%), and one choriocarcinoma (0.6%). According to the TNM classification, the cohort consisted of 52 (29.2%) pT1, 108 (60.7%) pT2, and 18 (10.2%) pT3 patients. Table 1 summarizes the histological subtypes of tumors and their corresponding stages following orchiectomy.

No significant difference in PIV scores was observed when comparing tumor histological types and laterality. Analysis of the relationship between PIV and TNM stage revealed a statistically significant increase in PIV scores with higher tumor T stages (p = 0.01) (Table 1). The mean PIV scores were significantly higher in patients with lymph node involvement and metastasis compared with those without (p = 0.01 and p = 0.04, respectively). The association between a history of retroperitoneal lymph node dissection (RPLND) and PIV was not statistically significant (p = 0.574). Analysis of the relationship between PIV scores and IGCCCG risk groups revealed a statistically significant increase in PIV values among higher risk categories (p = 0.01) (Table 2).

**Figure 1:**
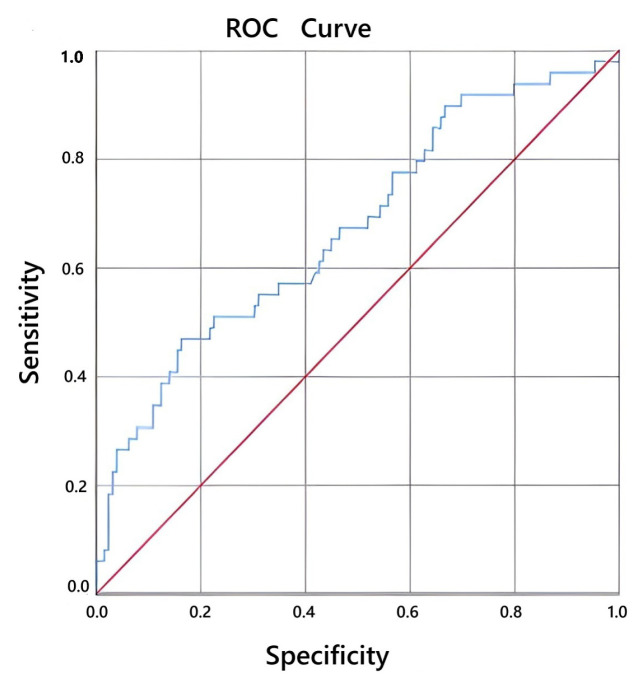
Evaluation of the relationship between PIV ratio score and retroperitoneal lymph node invasion.

**Figure 2:**
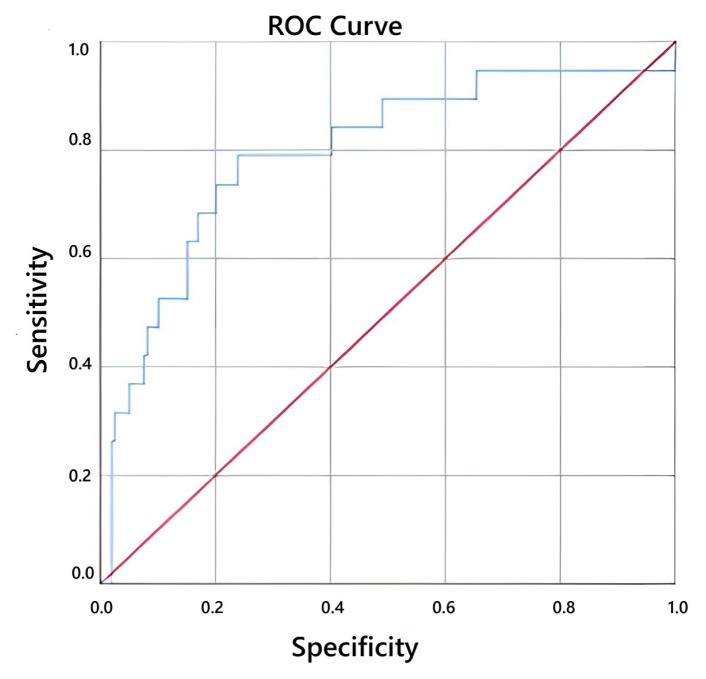
Evaluation of the relationship between PIV ratio score and metastasis.

The optimal cut-off value of the PIV score for predicting lymph node positivity was 555.5, with 75.5% sensitivity and 77.5% specificity as shown as Figure 1 (AUC = 0.802; 95% CI, 0.729–0.875; p < 0.001). The corresponding cut-off for predicting metastasis was 565.32, with 78.9% sensitivity and 76.1% specificity (AUC = 0.795; 95% CI, 0.676–0.915; p < 0.001) as shown as Figure 2 (Table 3).

## 4. Discussion

The present study investigated the relationship between PIV and the clinicopathological characteristics of testicular cancer. This particular aspect has not been previously researched. Our findings revealed that PIV can predict the stage, lymph node involvement and distant in germ cell testicular tumors. Pan-immune inflammation value is a new biomarker that reflects systemic inflammation status by combining four parameters with a high predictive rate [14].

A healthy immune system has a significant role in detecting and eliminating cancer cells. However, cancer cells have a favorable condition to evade apoptosis under immunosuppressive status. Besides that, some kind of tumoral cells might manipulate the immune system to their advantage to create an immunosuppressive microenvironment that facilitate their growth and spread [15]. With all these information, many studies have reported on the relationship between inflammation and cancer prognosis [2,3,10,16]. Based on this studies, PIV -a novel indicator combining four different hematological parameters- has been defined.

There are numerous studies that suggest PIV predicts prognosis in solid tumors. The increase in neutrophils and platelets, along with a decrease in lymphocytes, has previously been reported to be associated with poorer prognosis in cancer patients [17–19]. Therefore, PIV has been defined with combining more than one immune factors. PIV provides information about both tumoral microenvironment and the immune status [15].

The association between PIV and urological malignancies has been investigated in previous studies. Kayar et al. evaluated the relation between PIV and non-metastatic muscle invasive bladder cancer in 119 patients. They reported that high PIV value was associated with poorer overall survival (OS) and disease free survival (DFS). Additionally, it was stated that PIV is superior to systemic immune inflammation index (SII) and platelet-lymphocyte ratio in predicting DFS [11]. In another study about patients who underwent radical cystectomy, PIV and other inflammation biomarkers (SII, NLR) were compared. It was stated that high PIV value has a relationship with poor relapse free Survival and OS [20]. Zhu et al. investigated the success of systemic inflammation markers in predicting prostate cancer in patients with PSA levels between 4 and 20. They reported that higher PIV has highly effective in predicting prostate cancer (*p* = 0.001) [21].

The relationship between testicular cancer and inflammation markers has been previously investigated. Ekici et al. indicated that decreased hemoglobin, albumin, platelet, and lymphocyte scores (HALP) correlate with advanced disease and worse prognosis in individuals with testicular cancer [2]. Ilktac et al. investigated the association between NLR and testicular cancer. They reported that NLR is successful in predicting localized and non-localized disease [16]. In a review, it was reported that high SII values were associated with worse PFS and OS in patients with testicular cancer [22]. However, there are no prior studies investigating the relationship between PIV and testicular cancer.

Our findings reveal a statistically significant association between higher PIV scores and advanced tumor stage, presence of metastasis, presence of lymph node involvement in patients with testicular cancer. We found that PIV value higher than 555.5 was predictive for lymph node involvement. Additionally, we identified that when the PIV score exceeds 562.32 at the time of diagnosis, the likelihood of the patient being metastatic statistically increases.

We would like to emphasize that our study is the first of its kind. We think it is better to compare PIV with other immune markers such as NLR, PLR, SII in testicular cancer prognosis with subgroup analysis.

Our study has some limitations. First of all, our study was designed with a retrospective nature. Secondly, the study is based on data from a single tertiary center. A significant portion of the follow-up data of the patients were lacked after second postoperative year. Therefore, we are unable to provide cancer-spesific Survival or OS rates. We acknowledge that confounding factors cannot be entirely eliminated, as all indicators were obtained from peripheral blood samples of patients. Moreover, although our study did not reveal a significant difference between PIV and germ cell tumors, the absence of a subgroup analysis represents a limitation. We believe that the success of PIV might be better understood with multicentric, prospective studies.

## 5. Conclusion

This study is the first study that demonstrated a significant association between higher PIV scores and more advanced testicular tumors, presence of metastases. The PIV score, with its low cost-effectiveness, could serve as a prognostic tool for testicular tumor patients with high life expectancy. In this way, it can assist in stratifying risk and providing information about treatment management strategies at the time of diagnosis.

## Figures and Tables

**Table 1:** Association of PIV score with tumor laterality, metastasis and tumor subtype.

Parameters	n	%	PIV score Median (IQR)	p

Tumor laterality				
*Left*	73	59	454.21(100–4859.5)	0.28
*Right*	105	41	486.94(110.25–2784.2)	

Tumor subtype				
*Seminoma*	74	41.6	555.21(100.2–4859.3)	0.09
*Mixed germ cell tumor*	90	50.6	454.62 (100–2784.2)	
*Embryonal Carcinoma*	5	2.8	435.34(282.7–2164.1)	
*Teratoma*	7	3.9	346.6(177.93–1470.3)	
*Yolk Sac Tumor*	1	0.6	434.62	
*Choriocarcinoma*	1	0.6	446.25	

T stage				
*T1*	52	29.2	379.55(100–2822.77)	0.01
*T2*	108	60.6	472.5(100.19–4859.54)	
*T3*	18	10.2	843.6(321.34–2291.5)	

**Table 2:** Association of PIV score with RPLNI, T stage, RPLND and IUCC prognostic group.

Retroperitoneal lymph node invasion (RPLNI)				0.01
*Absence*	129	72.5	455.54 (100–4859.5)	
*Presence*	49	27.5	922.58(285.22–2164.12)	

Metastasis				0.04
*Absence*	159	89.3	447.27 (100–2822.7)	
*Presence*	19	10.7	638.74 (189.26–4859.54)	

Retroperitoneal lymph node dissection (RPLND)				0.574
*Absence*	168	94.4	456.53(100–4859.54)	
*Presence*	10	5.6	611.27(189.26–2784.21)	

IUCC prognostic group				0.01
*Stage 1*	122	68.5	446.14 (100–2822.77	
*Stage 2*	33	18.5	544.34 (189.26–4859.5)	
*Stage 3*	23	12.9	764.61 (285.22–2164.12)	

**Table 3:** Cut off values of PIV score in predicting RPLNI and metastasis.

	AUC (95%)	Cut-off value	P	Sensitivity	Specificity
RPLNI	0.802(0.729–0.875)	555.5	<0.001	0.755	0.775
Metastasis	0.795(0.676–0.915)	565.32	<0.001	0.789	0.761

## References

[1] Pfail J Santiago I Jang TL Paffenholz P EAU-YAU Penile and Testis Cancer Working Group. Contralateral Testicular Biopsy in Men with Testicular Cancer European Urology Focus 2024 10 3 370 372 10.1016/j.euf.2024.06.006 39095219

[2] Ekici O Akgun U Buyukdemirci E Avci S Caglayan V Association of hemoglobin, albumin, lymphocyte and platelet (HALP) score with testicular tumor aggressiveness and prognosis Urologia 2024 10 26 3915603241292199 10.1177/03915603241292199 39460560

[3] Sarejloo S Babadi S Khanzadeh S Salimi A Clark A Diagnostic and prognostic role of NLR in testicular cancer Exploration of Targeted Antitumor Therapy 2024 5 6 1177 1198 10.37349/etat.2024.00283 PMC11502077 39465013

[4] Park JS Kim J Elghiaty A Ham WS Recent global trends in testicular cancer incidence and mortality Medicine(Baltimore) 2018 97 37 e12390 10.1097/MD.0000000000012390 30213007 PMC6155960

[5] Jankovich M Jankovichova T Ondrus D Breza J Neutrophil to-lymphocyte ratio as a predictor of preoperative tumor staging in testicular germ cell tumors Bratislavske Lekarske Listy 2017 118 9 510 512 10.4149/BLL_2017_098 29061055

[6] Smith ZL Werntz RP Eggener SE Testicular Cancer: Epidemiology, Diagnosis, and Management Medicine Clinics of North America 2018 102 2 251 264 10.1016/j.mcna.2017.10.003 29406056

[7] Ling H Krassnig L Bullock MD Pichler M MicroRNAs in Testicular Cancer Diagnosis and Prognosis Urologic Clinicsof North America 2016 43 1 127 134 10.1016/j.ucl.2015.08.013 26614035

[8] Mantovani A Allavena P Sica A Balkwill F Cancer-related inflammation Nature 2008 24 7203 436 444 10.1038/nature07205 18650914

[9] Fuca G Guarini V Antoniotti C Morano F Morettao R The Pan-immune-inflammation Value is a new prognostic biomarker in metastatic colorectal cancer: results from a pooled-analysis of the Valentino and TRIBE first-line trials British Journal of Cancer2020; 123 3 403 409 10.1038/s41416-020-0894-7 PMC7403416 32424148

[10] Guven DC Sahin TK Erul E Kilickap S Gambichler T The Association between the Pan-immune-inflammation Value and Cancer Prognosis: a systematic review and Meta-Analysis Cancers (Basel) 2022 14 11 2675 10.3390/cancers14112675 35681656 PMC9179577

[11] Kayar R Bastug Y Tokuc E Topaktas R Akyurek EA Pan-immune-inflammation value as a prognostic tool for overall survival and disease-free survival in non-metastatic muscle-invasive bladder cancer International Urology and Nephrology 2024 56 2 509 518 10.1007/s11255-023-03812-w 37773579

[12] Yazgan SC Yekedüz E Utkan G Ürün Y Prognostic role of pan-immune-inflammation value in patients with metastatic castration-resistant prostate cancer treated with androgen receptor-signaling inhibitors The Prostate 2022 82 15 1456 1461 10.1002/pros.24419 35899494

[13] Patrikidou A Cazzaniga W Berney D Boormans J de Angst I European Association of Urology guidelines on testicular cancer: 2023 update European Urology 2023 84 3 289 301 10.1016/j.eururo.2023.04.010 37183161

[14] Liao W Li J Feng W Kong W Shen Y Pan-immune-inflammation value: a new prognostic index in epithelial ovarian cancer BMC Cancer 2024 24 1 1052 10.1186/s12885-024-12809-2 39187781 PMC11345988

[15] Yan S Gong X Liu R Jia X Prognostic significance of systemic pan-immune-inflammation value in locally advanced cervical cancer Frontiers in Oncology 2024 28 14 1492251 10.3389/fonc.2024.1492251 PMC11551031 39529824

[16] Ilktac A Dogan B Ersoz C Akcay M Akbulut H The relationship of neutrophil to lymphocyte ratio with testicular cancer International Brazilian Journal of Urology 2020 46 1 101 107 10.1590/s1677-5538.ibju.2019.0321 31851466 PMC6968911

[17] Teramukai S Kitano T Kishida Y Kawahara M Kubota K Pretreatment neutrophil count as an independent prognostic factor in advanced non-small-cell lung cancer: an analysis of Japan Multinational Trial Organisation LC00-03 European Journal of Cancer 2009 45 11 1950 1958 10.1016/j.ejca.2009.01.023 19231158

[18] Li L Wang J Meng S Li Z Huang Z Peripheral blood leukocytes and platelets serve as prognostic factors in breast cancer Cancer Biotherapyand Radiopharmaceuticals 2021 36 2 167 173 10.1089/cbr.2019.3032 32608994

[19] Quigley DA Kristensen V Predicting prognosis and therapeutic response from interactions between lymphocytes and tumor cells Molecular Oncology 2015 9 10 2054 2062 10.1016/j.molonc.2015.10.003 26607741 PMC5528725

[20] Russo P Palermo G Iacovelli R Ragonese M Ciccarese C Comparison of PIV and Other Immune Inflammation Markers of Oncological and Survival Outcomes in Patients Undergoing Radical Cystectomy Cancers (Basel) 2024 16 3 651 10.3390/cancers16030651 38339402 PMC10854772

[21] Zhu M Zhou Y Liu Z Jiang Z Qi W Diagnostic Efficiency of Pan-Immune-Inflammation Value to Predict Prostate Cancer in Patients with Prostate-Specific Antigen between 4 and 20 ng/mL Journal of Clinical Medicine 2023 19 3 820 10.3390/jcm12030820 PMC9917630 36769469

[22] Salazar-Valdivia FE Valdez-Cornejo VA Ulloque-Badaracco JR Hernandez-Bustamante EA Alarcón-Braga EA Systemic Immune-Inflammation Index and Mortality in Testicular Cancer: A Systematic Review and Meta-Analysis Diagnostics (Basel) 2023 22 5 843 10.3390/diagnostics13050843 PMC10000460 36899987

